# High serum concentrations of growth differentiation factor-15 and their association with Crohn’s disease and a low skeletal muscle index

**DOI:** 10.1038/s41598-022-10587-0

**Published:** 2022-04-21

**Authors:** Hiroyuki Yamamoto, Fuminao Takeshima, Masafumi Haraguchi, Yuko Akazawa, Kayoko Matsushima, Moto Kitayama, Kumi Ogihara, Maiko Tabuchi, Keiichi Hashiguchi, Naoyuki Yamaguchi, Hisamitsu Miyaaki, Hisayoshi Kondo, Kazuhiko Nakao

**Affiliations:** 1 Department of Gastroenterology and Hepatology, Graduate School of Biomedical Science, Nagasaki University, 1-7-1 Sakamoto, Nagasaki City, Nagasaki 852-8501 Japan; 2 Department of Internal Medicine, Nagasaki Prefecture Goto Central Hospital, Nagasaki, Japan; 3 Department of Tumor and Diagnostic Pathology, Atomic Bomb Disease Institute, Nagasaki University, Nagasaki, Japan; 4 Department of Endoscopy, Nagasaki University Hospital, Nagasaki, Japan; 5 Biostatistics Section, Division of Scientific Data Registry, Atomic Bomb Disease Institute, Nagasaki University, Nagasaki, Japan

**Keywords:** Biomarkers, Gastroenterology, Medical research

## Abstract

Sarcopenia comprises a low skeletal muscle index (SMI) and low muscle strength (MS) or low physical function. Many sarcopenia biomarkers have been reported. With Crohn’s disease (CD), a low SMI is predictive of intestinal complications. Therefore, many CD studies have reported that sarcopenia is defined by SMI alone. This study investigated the sarcopenia frequency by assessing the SMI and MS of Japanese patients with CD and biomarkers predicting a low SMI. We evaluated the SMI using a bioelectrical impedance analysis, handgrip strength, and C-reactive protein, albumin, interleukin-6, tumor necrosis factor-α, growth differentiation factor (GDF)-8, and GDF-15 levels as biomarker candidates for 78 CD patients at our hospital. Sarcopenia and a low SMI were observed in 7.7% and 42.3% of the patients, respectively. There was a significant difference in the GDF-15 levels of the low SMI group and normal group according to the multivariate analysis (P = 0.028; odds ratio [OR], 1.001; 95% confidence interval [CI] 1.000–1.002). When evaluated by sex, males exhibited a negative correlation between the GDF-15 level and SMI (Pearson’s r = − 0.414; P = 0.0031), and the multivariate analysis indicated a significant difference in the GDF-15 levels (P = 0.011; OR, 1.001; 95% CI 1.000–1.002). GDF-15 levels may indicate a low SMI with CD.

## Introduction

Sarcopenia was proposed by Rosenberg in 1989 as a term for age-related muscle loss^1^. Subsequently, the European Working Group on Sarcopenia in Older People classified age-related sarcopenia as primary sarcopenia and activity-related, disease-related, and nutrition-related sarcopenia as secondary sarcopenia^2^. Many research groups, such as the Asian Working Group for Sarcopenia (AWGS), defined the diagnostic criteria for sarcopenia as either low muscle strength (MS) or low physical function in addition to a low skeletal muscle mass index (SMI)^3^.

Young people with Crohn’s disease (CD), which is a debilitating disease of unknown cause, often experience abdominal pain, diarrhea, and weight loss. Curative treatment cannot be expected for CD. Medical treatment such as anti-tumor necrosis factor-alpha (TNF-α) antibody is the main treatment for CD; however, surgical treatment is required for intestinal complications such as severe stenosis and fistulas. Most previous studies of CD have reported that sarcopenia is often defined by SMI alone. A recent meta-analysis^4^ reported that 52% of patients had CD that was defined by the SMI. Bryant et al. demonstrated that the prevalence of sarcopenia defined by SMI and grip strength was 12% for patients with CD^5^. However, no study has demonstrated the prevalence of sarcopenia diagnosed using the appropriate definition for Japanese patients with CD.

The cumulative surgery rate for CD is approximately 50% at 10 years after onset^6^. The cumulative reoperation rates after intestinal resection are 22–28% and 30–40% at 5 years and 10 years after surgery, respectively^7^. Recurrence at the anastomotic site under endoscopic observation occurs frequently, with rates of 70–90% at 1 year after surgery^8,9^. Therefore, intestinal resection should be avoided as often as possible because CD is associated with early postoperative recurrence. Bamba et al. reported that sarcopenia defined by SMI is a predictor of intestinal resection with CD^10^. There have been several studies of the relationship between SMI and surgery and the association between a low SMI and postoperative complications with CD^11^.

Therefore, a low SMI is an important problem for CD patients who are considered for intestinal resection. The SMI was measured using a dual energy X-ray absorptiometry or bioelectrical impedance analysis (BIA); however, both require expensive measuring equipment. Computed tomography (CT) is also used, but it requires specific software and has the disadvantage of exposing the patient to radiation, thus making it difficult to perform many times. Therefore, there are few facilities where SMI can be measured.

Because various mechanisms contribute to sarcopenia, there have been reports of several biomarkers that can help diagnose sarcopenia. Franscesco et al. summarized previous studies of sarcopenia biomarkers according to their mechanism^12^. For example, albumin (ALB), which is behavior-mediated, is an indicator of nutritional status, and undernutrition can be an indicator of sarcopenia^13^. Growth differentiation factor (GDF)-8 and GDF-15 suppress muscle growth^14,15^. Interleukin (IL)-6 and TNF-α are inflammation-mediated, act as receptors in skeletal muscle, and promote muscle atrophy^16^. If a biomarker that predicts a low SMI with CD can be identified, then it will be useful for daily medical care.

This study is to first reveal the prevalence of “true sarcopenia” assessed using MS and the SMI of Japanese CD patients. Furthermore, it searched for biomarkers that could be indicators of a low SMI.

## Methods

### Study design and patients

We performed a cross-sectional study of 78 consecutive outpatients or inpatients with CD who visited the Department of Gastroenterology at Nagasaki University Hospital between August 2018 and July 2020. Our research protocol complied with the guidelines of the Declaration of Helsinki and was approved by the Nagasaki University Ethics Committee (approval no. 18121006). All patients provided informed written consent; if the patients were minors, then their parents provided consent. The diagnosis of CD was based on standardized criteria determined using the results of clinical assessment, endoscopy, small bowel radiography, and histology. The exclusion criteria for our study were severe CD or mobility difficulties that made it difficult to diagnose sarcopenia and the use of implantable medical devices. The inclusion criteria were CD, fulfillment of none of the aforementioned exclusion criteria, consecutive outpatient or inpatient treatment at our hospital during the observation period.

### Clinical and laboratory data

Data regarding age, body mass index (BMI), the CD Activity Index (CDAI), presence or absence of biological therapy or surgery, and disease duration were collected. Blood tests were performed and C-reactive protein (CRP), ALB, IL-6, TNF-α, GDF-8, and GDF-15 levels were measured as biomarkers. To measure GDF-8 and GDF-15 concentrations in serum, we used the GDF/Myostatin Quantikine enzyme-linked immunosorbent assay (ELISA) and Human GDF-15 Quantikine ELISA (R&D Systems, Minneapolis, MN, USA) according to the manufacturer’s instructions.

### Evaluation of the SMI, MS, and sarcopenia

During this study, sarcopenia was diagnosed using the assessment criteria provided by the 2019 Consensus Update on Sarcopenia Diagnosis and Treatment reported by the AWGS^3^. According to these criteria, sarcopenia was defined as a low SMI and low handgrip strength. Handgrip strength was measured using a Smedley handgrip dynamometer (TTM, Tokyo, Japan) while the participant was in a standing position. Two trials were performed for the right and left hands, and the two highest values were averaged and entered into the analysis. The cutoff values for low MS were 28 kg for males and 18 kg for females. The SMI was calculated using the BIA (InBody 770; InBody Japan, Tokyo, Japan)^17^; the sum of the skeletal muscle mass of the arms and legs was divided by the square of the individual’s height (kg/m^2^). The cutoff values for a low SMI were 7.0 kg/m^2^ for males and 5.7 kg/m^2^ for females.

If a CT scan was performed according to clinical needs within approximately 1 month after consent was obtained, then the cross-sectional area of the skeletal muscles (cm^2^) at the level of the third lumbar (L3) vertebra was measured using image analysis software (sliceOmatic V4.3; TomoVision, Magog, Quebec, Canada)^18^. The SMI was calculated based on the sum of this area divided by the square of the height (cm^2^/m^2^).

### Statistical analysis

Except for one patient, statistical analyses were performed using JMP Pro version 15.0 software (SAS Institute Japan, Tokyo, Japan). P < 0.05 was considered statistically significant for all tests. Furthermore, t tests or U tests were used to compare continuous variables, such as age, and the χ^2^ test was used to compare sex and other variables of the two groups. Correlation coefficients (Pearson correlation) were calculated to determine the correlation between the BIA and CT for measuring the SMI. For biomarkers that showed significant differences between the low SMI and normal SMI groups, a multivariate analysis adjusted for sex, age, and BMI was performed using a logistic regression analysis, and the P-value, odds ratios (ORs), and 95% confidence intervals (CIs) were calculated. We adjusted for age and BMI because aging is one factor associated with sarcopenia, and BMI is correlated with the SMI of irritable bowel disease patients^10^. Regarding the adjustment for sex, propensity score matching was performed using SAS software (version 9.4; SAS Institute, Inc., Cary, NC, USA). Additionally, we examined the Pearson correlations between biomarkers and SMI according to sex.

## Results

### Prevalence of sarcopenia and a low SMI

Of the 79 patients who participated in this study, 78 were included in the analysis set; those who did not participate in the grip strength test and BIA were excluded. The clinical characteristics of the patients are shown in Table 1. Among these patients, 64.1% were male, the median age was 42 years (range, 31.8–51 years), the median BMI was 19.9 kg/m^2^ (18.6–21.7 kg/m^2^), the median disease duration was 12.29 years (range, 6.44–19 years), and the median CDAI was 84.5 (range, 53.5–119). Sarcopenia confirmed by low MS and a low SMI was diagnosed in six patients (7.7%). Thirty-three patients (42.3%) had a low SMI.

**Table 1:** Patients’ clinical characteristics.

	Total (n = 78)	Male (n = 50)	Female (n = 28)	P-value
Age (years), median (IQR)	42 (31.8–51)	43 (33–51.3)	39 (29.5–48.5)	0.3
Body mass index (kg/m^2^), median (IQR)	19.9 (18.6–21.7)	20 (18.7–21.9)	19.4 (18.4–21.3)	0.24
Height (cm), median (IQR)	166 (159.0–173.8)	170.2 (166–174.8)	157.6 (152.3–162.0)	< 0.0001
Weight (kg), median (IQR)	55.5 (49.3–62.0)	58.2 (53.0–63.8)	49.2 (42.6–54.6)	< 0.0001
Grip strength (kg), median (IQR)	32.9 (25.8–40.8)	37.3 (32.7–42.3)	23.9 (19.8–26.8)	< 0.0001
Decreased handgrip strength (%)	7 (9.3)	6 (12)	1 (4)	0.26
SMI (kg/m^2^), median (IQR)	6.7 (6.0–7.4)	7.3 (6.7–7.5)	5.5 (5.1–6.1)	< 0.0001
Decreased SMI (%)	33 (42.3)	17 (34)	16 (57.1)	0.047
With sarcopenia (%)	6 (7.7)	5 (10)	1 (4)	0.31
Biological therapy (for CD) (%)	69 (88.5)	46 (92)	23 (82.1)	0.22
Disease duration (year), median (IQR)	12.29 (6.44–19)	14 (6.74–20.65)	11.25 (6.15–17.98)	0.27
CDAI, median (IQR)	84.5 (53.5–119)	83 (52–125)	86 (60.5–114.5)	0.62
CRP (mg/dL), median (IQR)	0.13 (0.05–0.35)	0.2 (0.06–0.37)	0.1 (0.04–0.32)	0.15
ALB (g/dL), median (IQR)	4 (3.7–4.4)	3.8 (3.5–4.3)	4.1 (3.9–4.4)	0.069
IL-6 (pg/mL), median (IQR)	1.64 (0.92–3.38)	1.96 (1.31–3.83)	1.10 (0.80–2.37)	0.0073
TNF-α (pg/mL), median (IQR)	8.49 (4.46–47.5)	9.46 (4.46–39.65)	8.4 (5.05–50.65)	0.76
GDF-8 (pg/mL), median (IQR)	3808 (3174.8–5282.6)	4142.7 (3310.6–6019.8)	3371.7 (2820.2–4061.3)	0.004
GDF-15 (pg/mL), median (IQR)	477.1 (296.3–1134.22)	795.1 (358.5–1494)	349.3 (282.6–831.8)	0.045

Data were analyzed using the U test or χ^2^ test.

*BMI* body mass index, *SMI* skeletal muscle mass index, *CDAI* Crohn’s disease activity index, *CRP* C-reactive protein, *ALB* albumin, *IL-6* Interleukin-6, *TNF-α* tumor necrosis factor-alpha, *GDF-8* growth differentiation factor-8, *GDF-15* growth differentiation factor-15, *IQR* interquartile range.

**Figure 1:**
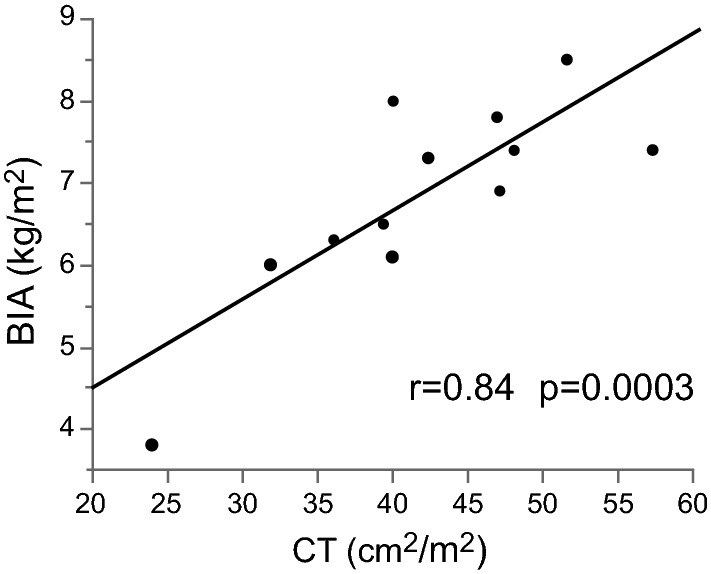
Correlation of the SMI with CT and the BIA. *SMI* skeletal muscle mass index, *BIA* bioelectrical impedance analysis, *CT* computed tomography.

We also investigated the correlation of SMI with CT and BIA for 13 patients who underwent CT within 1 month of BIA (Fig. 1). The Pearson correlation test showed a highly significant correlation (r = 0.83; P = 0.0003).

### Comparison of the sarcopenia group and the non-sarcopenia group

Characteristics of the patients who met the criteria for sarcopenia are shown in Table 2; 10% of males (5/50) and 3.6% of females (1/28) were diagnosed with sarcopenia. In the sarcopenia group, there was a tendency for long-term disease duration (20.02 ± 12.0 years; P = 0.14) and short stature (160.5 ± 8.3 cm; P = 0.13); however, the difference was not significant. Age, BMI, and CDAI were not significant indicators of sarcopenia.

**Table 2:** Comparison of the sarcopenia group and non-sarcopenia group.

	Sarcopenia (n = 6)	Non-sarcopenia (n = 72)	P-value
Male/female	5/1	45/27	0.31
Age (years), mean ± SD	48.5 ± 19.2	42.1 ± 12.9	0.26
BMI (kg/m^2^), mean ± SD	20.0 ± 3.48	20.5 ± 3.2	0.69
Height (cm), mean ± SD	160.5 ± 8.27	166.0 ± 8.4	0.13
Weight (kg), mean ± SD	51.8 ± 12.6	56.7 ± 10.9	0.31
Disease duration (year), mean ± SD	20.02 ± 12.00	13.65 ± 10.02	0.14
CDAI, mean ± SD	89.8 ± 39.9	95.7 ± 57.2	0.81

Data were analyzed using the t test or χ^2^ test.

*BMI* body mass index, *CDAI* Crohn’s disease activity index, *SD* standard deviation.

### Comparison of the low SMI group and normal SMI group

Because the number of patients with sarcopenia was too low to perform an analysis, we divided the patients into two subgroups according to the SMI value (low SMI group and normal SMI group). The characteristics of the two groups are shown in Table 3. There was a significantly higher number of females compared to males in the low SMI group (57.1%, P = 0.047). BMI (18.8 ± 2.3 kg/m^2^; P < 0.0001), height (162.5 ± 8.5 cm; P = 0.006), and weight (50.0 ± 8.8 kg; P < 0.0001) were significantly lower in the low SMI group. However, there were no significant differences in age, CDAI, or disease duration.

**Table 3:** Comparisons between the low SMI group and normal SMI group.

	Low SMI (n = 33)	Normal (n = 45)	P-value
Male/female	**17/16**	**33/12**	**0.047**
Age (year), mean ± SD	44.0 ± 14.6	41.6 ± 12.6	0.47
BMI (kg/m^2^), mean ± SD	**18.8 ± 2.3**	**21.6 ± 3.2**	** < 0.0001**
Height (cm), mean ± SD	**162.5 ± 8.5**	**167.8 ± 7.8**	**0.006**
Weight (kg), mean ± SD	**50.0 ± 8.8**	**61.0 ± 10.2**	** < 0.0001**
CDAI, mean ± SD	97.6 ± 59.5	93.4 ± 53.6	0.76
Disease duration (year), mean ± SD	15.9 ± 12.06	12.84 ± 8.57	0.21
CRP (mg/dL), mean ± SD	0.21 ± 0.31	0.39 ± 0.51	0.09
ALB (g/dL), mean ± SD	3.91 ± 0.56	3.92 ± 0.54	0.91
IL-6 (pg/mL), mean ± SD	2.69 ± 2.61	2.41 ± 2.07	0.6
TNF-α (pg/mL), mean ± SD	17.69 ± 20.45	28.63 ± 34.9	0.09
GDF-8 (pg/mL), mean ± SD,	4107.1 ± 1418.1	4244.9 ± 1558.2	0.7
GDF-15 (pg/mL), mean ± SD	**1511.0 ± 1646.3**	**688.2 ± 575.3**	**0.013**

Data was analyzed either by t test or χ^2^ test.

*BMI* body mass index, *CDAI* Crohn’s disease activity index, *CRP* C-reactive protein, *ALB* albumin, *IL-6* Interleukin-6, *TNF-α* tumor necrosis factor-alpha, *GDF-8* growth differentiation factor-8, *GDF-15* growth differentiation factor-15, *SD* standard deviation, *SMI* skeletal muscle mass index.

Significant values are in bold.

Regarding biomarkers, GDF-15 (1511.0 ± 1646.3 pg/mL; P = 0.013) was significantly higher in the low SMI group. The CRP level (0.21 ± 0.31 mg/dL; P = 0.09) and TNF-α level (17.69 ± 20.45 pg/mL; P = 0.09) tended to be lower in the low SMI group; however, the difference was not significant. No differences were observed for other biomarkers, including ALB, IL-6, and GDF-8. The multivariate analysis adjusted for sex, BMI, GDF-15 (Table 3), and age (Table 4) using a logistic regression analysis showed that the ORs and P-values were significant for sex (OR, 4.3; 95% CI 1.19–15.57; P = 0.026;), BMI (OR, 0.64; 95% CI 0.48–0.85; P = 0.002), and GDF-15 (OR, 1.001; 95% CI 1.000–1.002; P = 0.028). To confirm these results, we performed an additional multivariate analysis adjusted for age and BMI using propensity score matching to account for differences in GDF-15 according to sex and found significant differences in the OR and P-value for GDF-15 after matching (OR, 1.002; 95% CI 1.000–1.004; P = 0.021).

**Table 4:** Factors associated with a low SMI among CD patients.

Covariate	Univariate			Multivariate		
	P-value	OR	95% CI	P-value	OR	95% CI
Sex (female)	**0.049**	**2.59**	**1.001–6.69**	**0.026**	**4.3**	**1.19–15.57**
Age	0.43	1.014	0.98–1.05	0.83	1.006	0.96–1.06
BMI	**0.0007**	**0.64**	**0.50–0.83**	**0.002**	**0.64**	**0.48–0.85**
GDF-15	**0.015**	**1.001**	**1.000–1.001**	**0.028**	**1.001**	**1.000–1.002**

Data were analyzed using a univariate analysis and multivariate analysis and adjusted using a logistic regression analysis.

*BMI* body mass index, *CD* Crohn’s disease, *GDF-15* growth differentiation factor-15, *OR* odds ratio, *CI* confidence interval, *SMI* skeletal muscle mass index.

Significant values are in bold.

### Examination of biomarkers according to sex

Because sex affects the frequency of a low SMI or the value of some biomarkers, we examined the biomarkers associated with SMI according to sex. The correlations between the SMI and each biomarker are shown in Table 5. The value of GDF-15 (r = − 0.414; P = 0.0031) was negatively correlated with the SMI of males. CRP, ALB, IL-6, TNF-α, and GDF-8 levels were not correlated with the SMI. For females, the ALB value (r = 0.377; P = 0.048) was positively correlated with the SMI, but the IL-6 value (r = − 0.484; P = 0.012) was negatively correlated with the SMI. The CRP, TNF-α, GDF-8, and GDF-15 levels were not correlated with the SMI. Because GDF-15 levels in males and ALB and IL-6 levels in females were significantly correlated with SMI, we performed a multivariate analysis of these biomarkers and adjusted for age and sex by logistic regression analysis. For males (Table 6), a high GDF-15 level (OR, 1.001; 95% CI 1.000–1.002; P = 0.011) was an independent indicator of low SMI. The area under the receiver-operating characteristic curve was 0.755, the cutoff value was 940.11 pg/mL using Youden index, the sensitivity was 75%, and the specificity was 76%. We performed the same analysis for females and for males; however, the low ALB levels (OR, 0.35; 95% CI 0.019–6.23; P = 0.47) and high IL-6 levels (OR, 0.38; 95% CI 0.07–2.02; P = 0.26) did not significantly contribute to a low SMI.

**Table 5:** Pearson correlation with the SMI of male and females.

	Male		Female	
	r	P-value	r	P-value
CRP	0.0623	0.67	0.0637	0.75
ALB	0.132	0.37	**0.377**	**0.048**
IL-6	− 0.242	0.094	− **0.484**	**0.012**
TNF-α	0.163	0.262	− 0.011	0.96
GDF-8	0.153	0.29	0.0993	0.63
GDF-15	− **0.414**	**0.0031**	− 0.248	0.22

Data were analyzed by the Pearson product-moment correlation coefficient.

*CRP* C-reactive protein, *ALB* albumin, *IL-6* interleukin-6, *TNF-α* tumor necrosis factor-alpha, *GDF-8* growth differentiation factor-8, *GDF-15* growth differentiation factor-15, *SMI* skeletal muscle mass index.

Significant values are in bold.

**Table 6:** Factors associated with a low SMI among male CD patients.

Covariate	Univariate			Multivariate		
	P-value	OR	95% CI	P-value	OR	95% CI
Age	**0.02**	**1.06**	**1.01–1.12**	0.26	1.04	0.97–1.11
BMI	**0.021**	**0.76**	**0.57–0.997**	0.049	0.75	0.54–1.03
GDF-15	**0.0006**	**1.001**	**1.000–1.002**	**0.011**	**1.001**	**1.000–1.002**

Data were analyzed using a univariate analysis and multivariate analysis and adjusted by a logistic regression analysis.

*BMI* body mass index, *GDF-15* growth differentiation factor-15, *OR* odds ratio, *CI* confidence interval, *CD* Crohn’s disease.

Significant values are in bold.

## Discussion

This cross-sectional study demonstrated the frequency of sarcopenia using an appropriate definition and by incorporating assessments of muscle mass and strength. Among Japanese patients with CD, 42.3% had a low SMI and 7.7% had sarcopenia. In general, the peak grip strength is achieved at approximately 30 years of age; then, it declines with age older than 40 years^19^. We thought that the patient cohort, with a median age of 42 years, would have a grip strength that was close to the peak, and that only a small number of both males and females would meet the criteria for low grip strength. We had no data regarding the healthy controls who were the same age as the individuals in the patient cohort (approximately 30 years of age). However, the frequency of sarcopenia with CD for the patient cohort was approximately the same as that for healthy individuals older than 60 years, which was reported as 8.2% by a recent survey^20^. The frequency of a low SMI was significantly higher for females (57.1%; p = 0.047), indicating a sex difference. Although few studies have examined the SMI of CD patients according to sex, one study defined sarcopenia in terms of the SMI (i.e., low SMI in this study) and reported that the frequency was 56.4% for males and 81.4% for females with CD^21^. Although the criteria for low SMI differed between the previous study and our study, and although the percentage of females with a low SMI differed greatly, it was observed that more females had a low SMI compared to males in both studies. The reason for the significantly higher rate for females is unclear; however, it should be noted that our study applied the AWGS 2019 criteria for low SMI, which assume that elderly individuals would be included. Because the cutoff value is also based on elderly individuals, the cutoff value in our study may not have been appropriate. Additionally, young females may restrict their diets more often than males, which may have contributed to our findings.

Although CT is well-recognized as the gold standard investigative tool for the SMI, it exposes patients to radiation exposure and is costly. Moreover, CT results can be obtained only when the examination is necessary in clinical practice. Most previous studies of sarcopenia with CD used CT to estimate the SMI and were analyzed retrospectively. The groups in those studies were biased toward the patients who required CT examinations in clinical practice. During this study, we showed that the BIA measurements of the SMI were strongly correlated with CT-based estimates. Therefore, the BIA is also useful for measuring the muscle mass of patients with CD. The BIA should be actively used during future prospective studies of sarcopenia with CD because it is non-invasive and convenient. However, measuring the SMI requires special equipment such as CT or the BIA, which makes it difficult to examine the SMI in daily practice at general hospitals and clinics. If there is a biomarker that can predict SMI, then it should be able to be measured at any institution. This might help when determining nutritional therapy, for example. Some patients with CD require nutritional therapy, such as component nutrients. One systematic review showed that nutritional supplementation for sarcopenia improves muscle mass and strength^22^. Therefore, the prediction of a low SMI by biomarkers for patients with CD would be useful when considering the indications for nutritional therapy and rehabilitation nutrition.

GDF-15 is a member of the transforming growth factor-beta superfamily and is expressed at low concentrations in various organs such as the liver, kidneys, lungs, and other tissues under normal physiological conditions^23,24^. Elevated levels of inflammatory cytokines, oxidative stress, and hypoxia are known to elevate GDF-15 expression. In our study, GDF-15 was significantly higher with a low SMI, as shown by the multivariate analysis results (Table 4), which were similar when matched by sex. Therefore, GDF-15 could be an independent indicator of a low SMI. We also demonstrated that GDF-15 is negatively correlated with the SMI of male patients with CD and can be an indicator of a low SMI according to the multivariate analysis adjusted for age and BMI. However, there was no correlation between GDF-15 and the SMI for females. GDF-15 levels were higher in females than in males in their 30 s; however, they were higher in males than in females when they were in their 50s^25^. The population observed during our study was in their 30 s, when this change begins to occur, which may be why there was no significant difference in females. However, the most likely reason is that the number of females was smaller than the number of males. In the future, it would be desirable to accumulate more cases, especially those of females, for further evaluation, thereby possibly allowing us to find a correlation between the SMI and GDF-15 and other biomarkers in females. The inverse correlation between muscle mass and the circulating GDF-15 level is not specific to CD and has been observed in patients with chronic obstructive pulmonary disease^26^, pulmonary arterial hypertension^27^, and preoperative cardiovascular disease^28^. Moreover, an inverse association between GDF-15 and MS or muscle function has been observed in patients with cardiometabolic disease^29^ and cancer^30^ and in healthy community-dwelling adults^31^. In our study, there were two patients with a history of surgery for cancer (rectal cancer and thyroid cancer). Because their cancers were surgically resectable and had not recurred many years after surgery, we considered that the cancers had been cured and included these patients in the study. Although the exact mechanisms of serum GDF-15 elevation in CD patients with low SMI remain unknown, some evidence has suggested that GDF-15 may promote muscle wasting. It has been reported that glial cell-derived neurotrophic factor receptor alpha-like, the receptor for GDF-15, is present in the brainstem, and that the binding of GDF-15 leads to the loss of appetite and weight loss^32–35^. Additionally, several reports have suggested a direct catabolic effect of GDF-15 on muscle mass. Patel et al.^26^ demonstrated that local overexpression of GDF-15 leads to a reduction in the fiber size of the tibialis anterior muscle in mice. Furthermore, genetic loss of GDF-15 did not affect muscle wasting in transgenic mice characterized by mitochondrial stress-driven skeletal muscle atrophy^36^. GDF-15 treatment of C2C12 myotubes increased the mRNA expression of muscle atrophy-related genes, such as *MuRF-1* and *Atrogin*, and downregulated the expression of muscle microRNAs, such as miR-1, miR-133a, and miR-181a^37^. Further studies are needed to clarify the relationship between circulating GDF-15 levels and muscle wasting in patients with CD and sarcopenia-related outcomes.

Our study had several limitations. First, sarcopenia was evaluated according to MS and the SMI. The AWGS criteria for sarcopenia include a low MS or low physical function in addition to a low SMI^3^; however, we did not evaluate physical function (i.e., 6-m walking speed or sit-to-stand test) during this study. If we had evaluated the physical function as well as MS, then we might have been able to determine the sarcopenia rate for CD patients and comparatively analyze the sarcopenia and non-sarcopenia groups more accurately. Second, because our study was a single-center, cross-center study, the causal relationship between GDF-15 and the SMI of CD patients was unclear. Despite these limitations, to our knowledge, this study is the first to evaluate the association between the SMI and GDF-15 for CD patients. We hope that GDF-15 will be considered an indicator of a low SMI and therapeutic effects for patients with CD through the accumulation of additional case data.

In conclusion, 42.3% of Japanese patients with CD had a low SMI and 7.7% were diagnosed with sarcopenia. A low SMI was associated with GDF-15 in CD patients. Therefore, GDF-15 may be a predictive biomarker for a low SMI and indicate a poor prognosis for CD patients.

## Data Availability

All data generated or analyzed in this study are included in this published article.
